# Fos Facilitates Gallid Alpha-Herpesvirus 1 Infection by Transcriptional Control of Host Metabolic Genes and Viral Immediate Early Gene

**DOI:** 10.3390/v13061110

**Published:** 2021-06-09

**Authors:** Zhitao Wang, Yangyang Qiao, Zhijie Chen, Yumeng Liang, Lu Cui, Yanhui Zhang, Xuefeng Li, Li Xu, Ping Wei, Shengwang Liu, Hai Li

**Affiliations:** 1 State Key Laboratory of Veterinary Biotechnology, National Poultry Laboratory Animal Resource Center, Harbin Veterinary Research Institute, the Chinese Academy of Agricultural Sciences, Harbin 150069, China; lydxwzt@163.com (Z.W.); yyqiao2010@163.com (Y.Q.); ws103czj@163.com (Z.C.); m1584702@163.com (Y.L.); cuilu099@163.com (L.C.); yhzhang10756@163.com (Y.Z.); mi942484673@163.com (X.L.); xxx1244397128@163.com (L.X.); 2 College of Veterinary Medicine, Northeast Agricultural University, Harbin 150030, China

**Keywords:** Gallid alpha-herpesvirus 1, Fos, Jun, metabolic genes, *ICP4*

## Abstract

Gallid alpha-herpesvirus 1, also known as avian infectious laryngotracheitis virus (ILTV), continues to cause huge economic losses to the poultry industry worldwide. Similar to that of other herpesvirus-encoded proteins, the expression of viral genes encoded by ILTV is regulated by a cascade, and the underlying regulatory mechanism remains largely unclear. The viral immediate-early (IE) gene *ICP4* plays a prominent role in the initiation of the transcription of early and late genes during ILTV replication. In this study, we identified AP-1 as the key regulator of the transcription of ILTV genes by bioinformatics analysis of genome-wide transcriptome data. Subsequent functional studies of the key members of the AP-1 family revealed that Fos, but not Jun, regulates ILTV infection through AP-1 since knockdown of Fos, but not Jun, by gene silencing significantly reduced *ICP4* transcription and subsequent viral genome replication and virion production. Using several approaches, we identified *ICP4* as a bona fide target gene of Fos that regulated Fos and has Fos response elements within its promoter. Neither the physical binding of Jun to the promoter of *ICP4* nor the transcriptional activity of Jun was observed. In addition, knockdown of Fos reduced the transcription of *MDH1* and *ATP5A1*, genes encoding two host rate-limiting enzymes essential for the production of the TCA intermediates OAA and ATP. The biological significance of the transcriptional regulation of *MDH1* and *ATP5A1* by Fos in ILTV infection was supported by the fact that anaplerosis of OAA and ATP rescued both *ICP4* transcription and virion production in infected cells under when Fos was silenced. Our study identified the transcription factor Fos as a key regulator of ILTV infection through its transcription factor function on both the virus and host sides, improving the current understanding of both avian herpesvirus–host interactions and the roles of AP-1 in viral infection.

## 1. Introduction

Gallid alpha-herpesvirus 1 (GaHV-1), formerly known as avian infectious laryngotracheitis virus (ILTV), belongs to the genus *Iltovirus*, the subfamily *Alphaherpesvirinae*, and the family *Herpesviridae* [1]. ILTV is shed in upper respiratory secretions and is easily transmitted by inhalation or mechanically transmitted by fomites and people. Infection with ILTV causes acute lytic infections of the upper respiratory tract in chickens and establishes latency in the trigeminal ganglia; it has an overall mortality rate of 5–70% and thus causes immense economic losses in the poultry industry worldwide [2,3]. Currently, there is no cure for infection with the virus, and the control of outbreaks mainly depends on vaccination. Although live-attenuated vaccines provide avians with protection against ILTV, the residual virulence of vaccine strains has been widely reported. Therefore, novel strategies are needed to improve the current control of ILTV outbreaks.

Viruses encoding a limited number of proteins rely on host factors to replicate in infected cells [4]. Host genes are crucial for the viral life cycle, including attachment and entry, viral gene transcription and genome replication, and virion assembly and egress [5]. Host–virus interactions are important for the successful replication and persistence of viruses in infected cells, the spread of viruses between host cells, and the final outcome of pathologies [6,7,8]. Thus, knowledge of interactions between viral and host factors may provide valuable information for developing novel antiviral strategies. Current studies on the interaction between avian herpesvirus and the host have mainly focused on Marek’s disease virus (MDV) [9,10]; similar studies on ILTV are limited.

Activator protein 1 (AP-1) is an intracellular ubiquitously expressed transcription factor (TF) that is comprised of proteins of the Jun and Fos families. Since the first verification of their roles as a DNA-binding proteins in 1987, members of the AP-1 family have been found to be a key modulator of the pathogenesis of viruses, including herpesviruses, through regulation of a wide range of biological processes, such as viral infection, replication, latency, and viral-associated cell transformation [11,12]. The viral protein ORF45 of Kaposi’s sarcoma-associated herpes virus (KSHV) increases viral transcription by promoting the direct interaction of Fos with viral promoters during KSHV lytic replication [13]. AP-1 acts as a regulator of the replication of cytomegalovirus (CMV) by binding to the promoter of the CMV major immediate early (IE) gene to trigger viral lytic replication [14]. The transcriptional activity of AP-1 has been found to be increased in herpes simplex virus type 2 (HSV-2)-infected cells [15]. In addition, an MDV protein, Meq, has been found to be similar to AP-1 family transcription factors and forms a stable heterodimer with host c-Jun to cause T-cell lymphomas [10]. However, no genes resembling the Meq gene have been found in the ILTV genome, and whether AP-1 members play any role in the life cycle of ILTV remains unknown.

In this study, we identified Fos as a key host regulator of ILTV infection through its TF function on both the virus and host sides, which involves regulation of the transcription of the viral IE gene *ICP4* and host metabolic genes.

## 2. Materials and Methods

### 2.1. Cell Culture and Transfection 

Chicken fibroblast (DF-1) cells and chemically immortalized leghorn male hepatoma (LMH) cells were maintained in Dulbecco’s modified Eagle’s medium (DMEM) supplemented with 10% fetal bovine serum (FBS), 100 U/mL penicillin and streptomycin and 2 mM L-glutamine. All cells were incubated at 37 °C in 5% CO_2_. For functional validation experiments, 5.0 μM ATP (Selleck, Houston, TX, USA, S1985) and 10 μM oxaloacetic acid (OAA, Sigma Aldrich, St. Louis, MO, USA, O7753) were added to the medium according to specific requirements. DF-1 and LMH cells were transfected with Lipofectamine 2000 (Invitrogen, Carlsbad, CA, USA) as recommended by the manufacturer.

### 2.2. Virus Preparation

The virulent ILTV-LJS09 strain (GenBank Accession No. JX458822) was stored at the Harbin Veterinary Research Institute of CAAS. When this strain is propagated in LMH cells, clear CPEs are observed [16,17]. ILTV-EGFP was constructed by replacing the *US9* gene of ILTV-LJS09 with the EGFP coding sequence as described previously [18].

### 2.3. Plasmids

A pCAGGS-HA expression plasmid containing an HA tag at the N-terminus was kindly provided by Harbin Veterinary Research Institute (Harbin, China). A firefly luciferase reporter plasmid (pGL3-Basic vector) and control *Renilla* luciferase plasmid (pRL-TK) were purchased from Promega. The coding sequences of chicken full-length Jun and Fos were amplified from cDNA of LMH cells, and the primer sequences are listed in Table 1. The PCR product was purified and digested with *XhoI* and *KpnI*. To generate the pCAGGS-HA-Jun and pCAGGS-HA-Fos plasmids, the digested or gel-purified PCR product was cloned into the pCAGGS-HA vector with T4 DNA ligase (NEB, Ipswich, MA, USA).

The *ICP4* DNA sequence was obtained from the sequenced ILTV genome in the NCBI database. Then, a promoter region (1 kb) in this gene upstream from the transcription start site was selected. The pGL3-Basic vector was digested with *XhoI* and *KpnI* for linearization. For the construction of a pGL3-*ICP4*-Luc plasmid containing the *ICP4* gene promoter, we prepared oligonucleotides (see Table 1). The linearized pGL3-basic vector and each promoter were linked using the In-Fusion cloning system (Takara, Dalian, China) according to the manufacturer’s instructions.

All PCR products were amplified with KOD-Plus-Neo (TOYOBO, Osaka, Japan) and all DNA constructs used in this study were verified by sequencing.

### 2.4. Western Blot Analysis

Western blotting was performed strictly according to previously described procedures [19]. Briefly, cells were washed with ice-cold PBS and soluble proteins were extracted with cell lysis buffer (100 mM Tris-HCl (pH = 8), 150 mM NaCl, 1% NP-40, phosphatase and protease inhibitor cocktail tablets (Abcam, Shanghai, China)) according to the manufacturer’s protocol. The protein concentration of each sample was determined by a BCA Kit (Beyotime, China). An equal amount of protein was separated by SDS-PAGE and transferred onto a nitrocellulose membrane (Millipore). The membrane was then blocked with 5% non-fat milk for 2 h at room temperature and incubated with primary antibodies overnight at 4 °C. Antibodies were obtained from Sigma-Aldrich (HA, # H9658) and Proteintech (Wuhan, China) (β-actin, # 60008-1-Ig). Signals were visualized using infrared imaging systems and quantified using ImageJ software v 1.0.20 (Odyssey CLX, LiCor Biosciences, Lincoln, NE, USA).

### 2.5. RNA Interference

Short interfering RNAs (siRNAs) that specifically recognized sequences in Fos mRNA (NM_205508; siFos, 5′-GGA UCC GCC GGG AGA GGA A-3′ and 5′-CCG ACA CUC UGC AGG CGG A-3′), Jun mRNA (NM_001031289; siJun, 5′-CCA GCA ACG GGU UAA UCA C-3′ and 5′-GAA CGU UAC CGA CGA GCA A-3′), and a control siRNA (siControl, 5′-UUC UCC GAA CGU GUC ACG UTT-3′) with no specific target site in chicken were used (Sigma). Subconfluent LMH cells seeded in 24-well plates were transfected with 5 pmol pooled siRNAs using Lipofectamine RNAiMAX (Invitrogen, Carlsbad, CA, USA) according to the manufacturer’s instructions. The siRNA-transfected cells were incubated for 24 h and then infected with ILTV at an MOI of 1. The cells were further incubated for 12 h and RNA was harvested.

### 2.6. Quantitative Real-Time PCR (qRT-PCR)

Total RNA was extracted from cells using TRIzol reagent (Takara, Dalian, China). The quality and concentration of total RNA were measured using a micro-volume spectrophotometer (Implen GmbH, Munich, Germany). A One-Step SYBR PrimeScript RT-PCR Kit (Takara, Tokyo, Japan) was used for qRT-PCR according to the manufacturer’s instructions. β-Actin was used as an endogenous control. The data were calculated with the 2^−ΔΔCT^ method and the results are presented as the log_2_ fold change. Genomic DNA was extracted using the AxyPrep Body Fluid Viral DNA/RNA Miniprep Kit (Axygen, Union City, CA, USA) according to the manufacturer’s instructions. Absolute qRT-PCR was performed using Luna Universal qPCR Master Mix (NEB, Ipswich, MA, USA) following the manufacturer’s protocol. Standards for absolute qRT-PCR were prepared by cloning the PCR products of the *gC* genes of ILTV into the pMD18-T plasmid (Takara, Shiga, Japan) according to the manufacturer’s instructions. At least three independent experiments were performed, with three technical replicates for each reaction. The primer sequences are listed in Table 2.

### 2.7. Chromatin Immunoprecipitation (ChIP) Assays

ChIP experiments were conducted as previously described [20] with some minor modifications. LMH cells were fixed in 1% formaldehyde for 10 min and then with 0.125 M glycine. Each ChIP experiment was performed with sheared chromatin samples from LMH cells (5 × 10^6^ cells) using 5 μg of one of the following antibodies: anti-HA (Sigma-Aldrich, St. Louis, MO, USA, mouse, # H9658) or isotype control IgG1 (Cell Signaling, Beverly, MA, USA, mouse, 5415). Protein A/G PLUS–agarose beads were used for pull-down according to the manufacturer’s instructions (Santa Cruz Biotechnology, Santa Cruz, CA, USA). The immunoprecipitated DNA was purified using a QIAquick PCR Purification Kit (QIAGEN, Valencia, CA, USA). The *ICP4* promoter region was detected by qRT-PCR using promoter DNA-specific primers with a Bio-Rad CFX96 instrument. Each reaction was performed in triplicate. The primers used for the ChIP-qPCR assay are shown in Table 3.

### 2.8. Luciferase Assays

LMH cells at 80–90% confluence were transfected in 48-well plates with 100 ng of pGL3-Basic vector, 2 ng of pRL-TK, and 100 ng of effector plasmid vector or effector plasmid expressing an Fos gene using Lipofectamine 2000 (Invitrogen, Carlsbad, CA, USA). The cells were lysed and subjected to luciferase assays using the Dual-Luciferase Reporter Assay System (Promega, Madison, WI, USA) 24 h after transfection according to the manufacturer’s instructions. The luciferase activity was determined with a Multiscan Spectrum (Enspire, PerkinElmer, Waltham, MA, USA). The relative luciferase activity was calculated by dividing the firefly luciferase activity by the *Renilla* luciferase activity. Three independent transfection experiments were performed.

### 2.9. Viral Quantitation

LMH cells were infected with ILTV at an MOI of 0.1 or 1. The indicated MOI was selected according to the number of cells to be infected and the estimated number of infectious particles based on the number of plaque-forming units detected in LMH cells. The level of virus replication was determined using plaque assays and ILTV-specific qPCR assays as previously described [18]. To determine the total level of viral replication, both cell-associated viruses and viruses released into the supernatant were collected for virus quantification. The cells were lysed via three rounds of freezing–thawing.

### 2.10. Bioinformatic Analysis

The transcription factors (TFs) of the differentially expressed genes (DEGs) were predicated using oPOSSUM v 3.0 (University of British Columbia, Vancouver, BC, Canada; http://opossum.cisreg.ca (accessed on 1 September 2012)) and human and mouse TF databases. The parameters were as follows: conservation cut-off = 40%; matrix score threshold = 85%; results sorted by Fisher score; amount of upstream/downstream = 5000/5000. Protein interaction information was acquired using the STRING database (http://www.string-db.org/ (accessed on 17 October 2020)). Cytoscape software was applied to visualize the protein interaction networks. The selection criteria were as follows: confidence score ≥ 0.4 and maximum number of interactors = 10.

KEGG pathway enrichment analyses were performed using DAVID to determine the functions of the DEGs. A *p* value < 0.05 and a count > 5 were considered to indicate statistical significance. The KEGG pathways were visualized in a bubble diagram with the ggplot2 package in R [21].

The *ICP4* gene promoter locus was obtained from the GenBank database. The 1-kb region upstream of the *ICP4* gene transcriptional start site was selected for promoter transcription factor binding site (TFBS) analysis. The *ICP4* promoter binding site position frequency matrix (PFM) was retrieved from Jaspar database version 2020 (http://jaspar.genereg.net/ (accessed on 22 January 2021)).

RNA sequencing raw data were uploaded to the NCBI database with accession number GSE138648.

### 2.11. Statistical Analysis

Data analyses was mainly performed with SPSS 13.0 (SPSS Inc., Chicago, IL, USA) and GraphPad Prism 7.0 software (GraphPad, Inc., San Diego, CA, USA). The significance of differences between two groups was determined by unpaired *t*-tests, and the significance of differences between multiple groups was determined by ANOVA. *p* < 0.05 was considered statistically significant.

## 3. Results

### 3.1. Genome-Wide Gene Expression Analysis Reveals AP-1 as a Central Modulator of Host Molecular Events upon ILTV Infection

**Figure 1:**
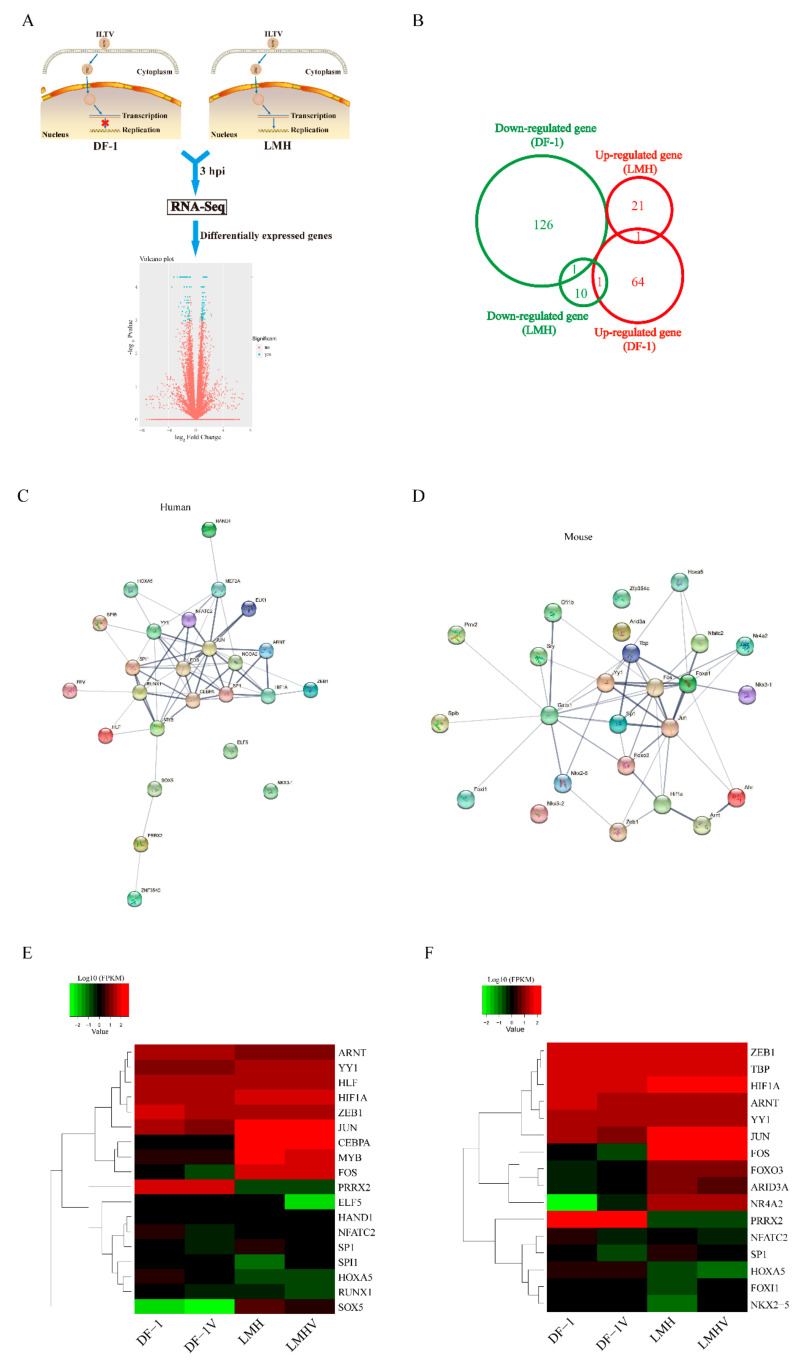
Bioinformatics analysis reveals AP-1 as a central modulator of host molecular events upon ILTV infection. (**A**) Schematic diagram showing the experimental design of the genome-wide transcriptome analysis. The ILTV-susceptible but nonpermissive chicken cell line DF-1, which only supports transient viral gene transcription, and the ILTV-permissive chicken cell line LMH were used as the models. The DEGs identified in LMH cells and DF-1 cells three hours after ILTV infection based on RNA-Seq were further analyzed. Volcano plot showing the log_2_ fold change values (X axis) by −log_10_ corrected *p*-values (Y axis) for all genes. The genes that were significantly altered (*p* < 0.01, |log_2_FC| > 1) are indicated as green dots. (**B**) Venn diagram representing the intersections of genes significantly regulated by ILTV infection in DF-1 cells and LMH cells (*p* < 0.05). Upregulated and downregulated genes are indicated in red and green, respectively. (**C**,**D**) Analysis of functional interactions between the TFs predicted by the significantly expressed genes reveals Fos and Jun as the most promising central modulators of the molecular network in ILTV-infected cells. (**C**) Human and (**D**) mouse TF interaction networks were generated based on the STRING database. Each node represents a TF, and each edge represents an interaction. (**E**,**F**) Cluster analysis of the TFs presented in (**C**,**D**) was performed based on RNA-Seq data (**E**: human database; **F**: mouse database). In the heatmap, each row or column represents one gene or sample, respectively. The color bars represent the log_2_ expression levels (FPKM, fragments per kilobase of exon per million fragments mapped) of each gene.

Our previous work using the ILTV-susceptible but nonpermissive chicken cell line DF-1 and the ILTV-permissive chicken cell line LMH as models revealed a significant shift in the transcription pattern of host genes in DF-1 cells but not LMH cells upon ILTV infection [22], which might cause transient viral gene transcription in DF-1 cells (Figure 1A). To explore the molecular mechanism underlying the distinct regulation of viral transcription in LMH cells and DF-1 cells, global transcriptome analysis of DF-1 and LMH cells at the early stage of ILTV infection was conducted; 193 differentially expressed genes (DEGs) were identified in DF-1 cells, but only 34 DEGs were identified in LMH cells upon ILTV infection. Among these DEGs, only two genes were commonly regulated between the two cell lines (Figure 1B). To further examine the mechanism of transcriptional regulation by ILTV infection in DF-1 cells, we performed transcription factor (TF) prediction using oPOSSUM v 3.0 (University of British Columbia, Vancouver, BC, Canada; http://opossum.cisreg.ca (accessed on 1 September 2012)) based on human and mouse TF databases. The top 25 TFs based on Fisher’s score were used for protein interaction analysis with the STRING database [23] to determine the central modulator of the molecular network in DF-1 cells after ILTV infection among the predicted TFs. Both analyses based on human or mouse databases revealed that Fos and Jun (AP-1) were located at the center of the network and had the highest numbers of connections (Figure 1C,D). In addition, hierarchical clustering analysis of the transcription profiles of these TFs in LMH cells and DF-1 cells upon ILTV infection based on RNA-seq data revealed that the expression levels of both Fos and Jun were significantly lower in DF-1 cells than in LMH cells regardless of infection status (Figure 1E,F). Thus, these findings suggest that AP-1 is a potential central modulator of the persistent transcription of viral genes.

### 3.2. Fos But Not Jun Is the Key Host Determinant of Viral Gene Transcription and Subsequent Viral Replication of ILTV

**Figure 2:**
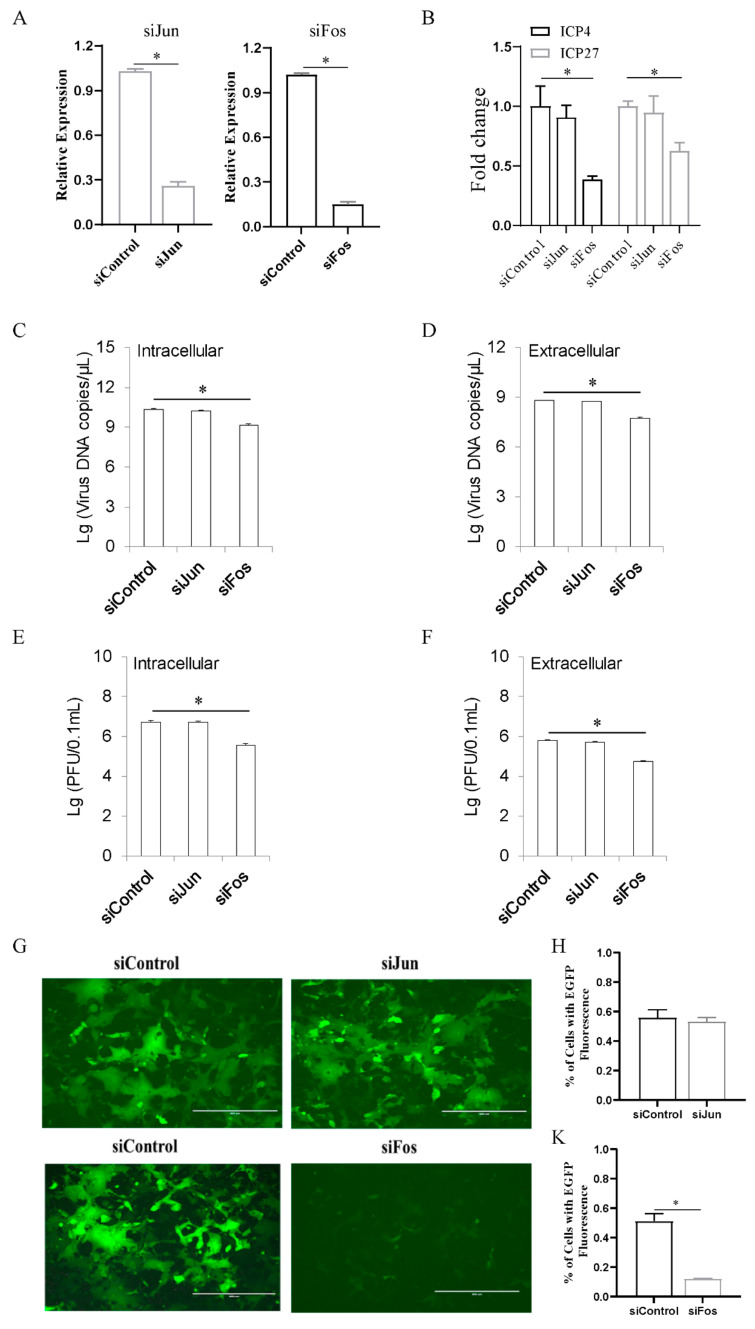
Fos is the key host determinant of ILTV replication in LMH cells. LMH cells were transiently transfected with Fos siRNA, Jun siRNA or negative control (NC) siRNA for 24 h. (**A**) The knockdown efficiencies of Fos siRNA and Jun siRNA were analyzed by qRT-PCR. (**B**) The transcription levels of *ICP4* and *ICP27* were quantitated 12 h after ILTV infection using qRT-PCR. (**C**,**D**) At 48 hpi, both the intracellular (**C**) and extracellular (**D**) viral genome copy numbers were determined by ILTV-specific qPCR. (**E**,**F**) The number of intracellular (**E**) and extracellular (**F**) infectious virions was quantified by plaque assays at 48 hpi. (**G**–**K**) LMH cells were transfected with Fos siRNA or Jun siRNA for 24 h before infection with ILTV-EGFP virus at an MOI of 1. (**G**) EGFP fluorescence (green) was observed by fluorescence microscopy at 36 hpi. The scale bars indicate 300 μm. (**H**,**K**) Quantification of the area of EGFP-positive LMH cells in each region. The percentage of LMH cells expressing EGFP in each field. The data are presented as the mean ± SD (*n* = 3). *, *p* < 0.05.

To elucidate the functional role of AP-1 in ILTV replication, Jun or Fos was knocked down in LMH cells prior to ILTV infection using siRNA. The knockdown efficiencies of the siRNAs were determined by qRT-PCR (Figure 2A). Knockdown of Fos in LMH cells significantly decreased the transcription of both the IE gene *ICP4* and the E gene *ICP27* at 12 h post infection (hpi), as determined by qRT-PCR (Figure 2B), while no significant effect of Jun on the transcription of either viral gene was observed, suggesting that Fos but not Jun plays a regulatory role in the ILTV gene transcription. Consistent with the effect of Fos on viral transcription, subsequent viral genome replication and virion production were also significantly reduced by Fos knockdown, as determined by analysis of intracellular and extracellular viral DNA (Figure 2C,D) and infectious virions (Figure 2E,F) in LMH cells at 48 hpi using ILTV-specific qRT-PCR and plaque assays, respectively. Our findings were further confirmed by the detection of EGFP signal in LMH cells infected with an ILTV strain expressing enhanced green fluorescent protein (EGFP) that we generated previously [18]; knockdown of Fos, but not Jun, significantly reduced the EGFP signal of ILTV-EGFP in LMH cells at 36 hpi (Figure 2G). Quantitative data for the area of EGFP-positive cells are presented in Figure 2H,K. These data revealed that Fos plays vital roles in ILTV IE gene transcription, DNA replication, and virion production.

### 3.3. Silencing of Fos Suppresses the Expression of Host Metabolic Genes

**Figure 3:**
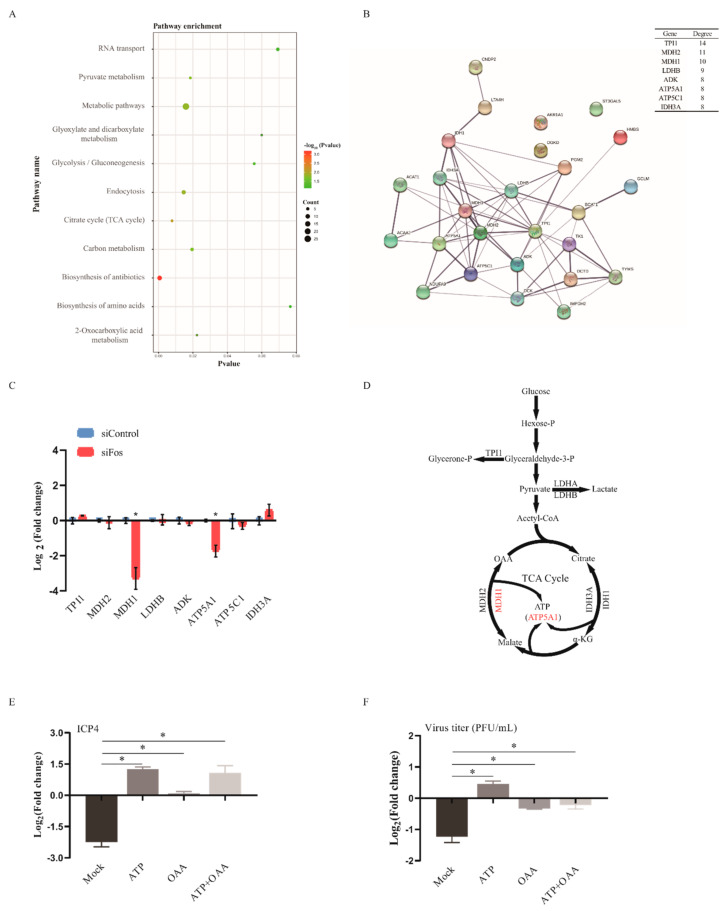
Effects of Fos silencing on metabolic genes in ILTV-infected LMH cells. (**A**) KEGG pathway enrichment analysis of the DEGs in DF-1 cells. The *X*-axis represents the *p*-values, the *Y*-axis represents the pathway name, and the color represents the −log_10_ (*p* value); the size of the point represents the number of genes. (**B**) The interaction network of differentially expressed metabolic genes visualized by STRING. The confidence parameter was set as 0.400, and the lines between genes represent possible interactions. The top 8 key nodes were arranged in order of their functional connectivity to other genes in the network. (**C**) LMH cells were transfected with control siRNA or Fos siRNA for 24 h and then infected with ILTV for 12 h at an MOI of 1. The transcription levels of the top 8 key metabolic genes were determined by qRT-PCR. (**D**) Schematic diagram representing the positions of these top key metabolic genes in metabolic pathways. (**E**,**F**) LMH cells were transfected with control siRNA or Fos siRNA for 24 h and infected with ILTV at an MOI of 1. (**E**) The transcription level of *ICP4* was assayed by qRT-PCR in LMH cells with or without pretreatment with ATP (5.0 μM) and/or OAA (10 μM) at 12 hpi with ILTV. (**F**) Viral titer was determined by plaque assay in LMH cells with or without pretreatment with ATP (5.0 μM) and/or OAA (10 μM) at 48 hpi (ILTV, MOI = 1). Mock refers to cells without added ATP and/or OAA. Each value represents the ratio of the value in the siFos group to that in the control siRNA group. All data were normalized to mock using the 2^−ΔΔCt^ method and are presented as the mean ± SD (*n* = 3). *, *p* < 0.05.

Next, we conducted pathway enrichment analysis of the DEGs in DF-1 cells upon ILTV infection. Consistent with our previous finding [22], ILTV infection mainly altered host metabolic processes in DF-1 cells, as presented in Figure 3A. Construction of a metabolic regulatory network of metabolic DEGs was performed with STRING. Eight hub genes, which may play essential roles in biological networks [24], were identified and considered the key metabolic genes causing the shift in the metabolic network in DF-1 cells upon ILTV infection (Figure 3B). Then, the effects of Fos on the expression of these hub metabolic genes were assessed by qRT-PCR, which revealed significant reductions in the transcription levels of *MDH1* and *ATP5A1* upon Fos silencing (Figure 3C). As shown in Figure 3D, NAD(H)-dependent malate dehydrogenase (MDH) catalyzes the reversible oxidation of L-malate to oxaloacetate (OAA), which plays critical roles in the tricarboxylic acid (TCA) cycle [25], and *ATP5A1*, a crucial part of complex V, is the rate-limiting enzyme of the synthesis of ATP from ADP and inorganic phosphate [26]. Our previous study revealed insufficient production of both OAA and ATP in DF-1 cells upon ILTV infection [22]. It is possible that Fos regulates ILTV replication by maintaining host OAA and ATP supplies via its transcriptional control of *MDH1* and *ATP5A1*. This hypothesis was verified by the fact that anaplerosis of ATP and OAA completely rescued the reduction in *ICP4* transcription (Figure 3E) and the subsequent reduction in virion production (Figure 3F) induced by Fos knockdown in LMH cells. Thus, Fos knockdown may repress ILTV transcription and virion production through its transcriptional control of host cellular metabolism.

### 3.4. Fos Overexpression Has No Effect on Either ILTV Replication or the Transcription of Metabolic Genes in DF-1 Cells

**Figure 4:**
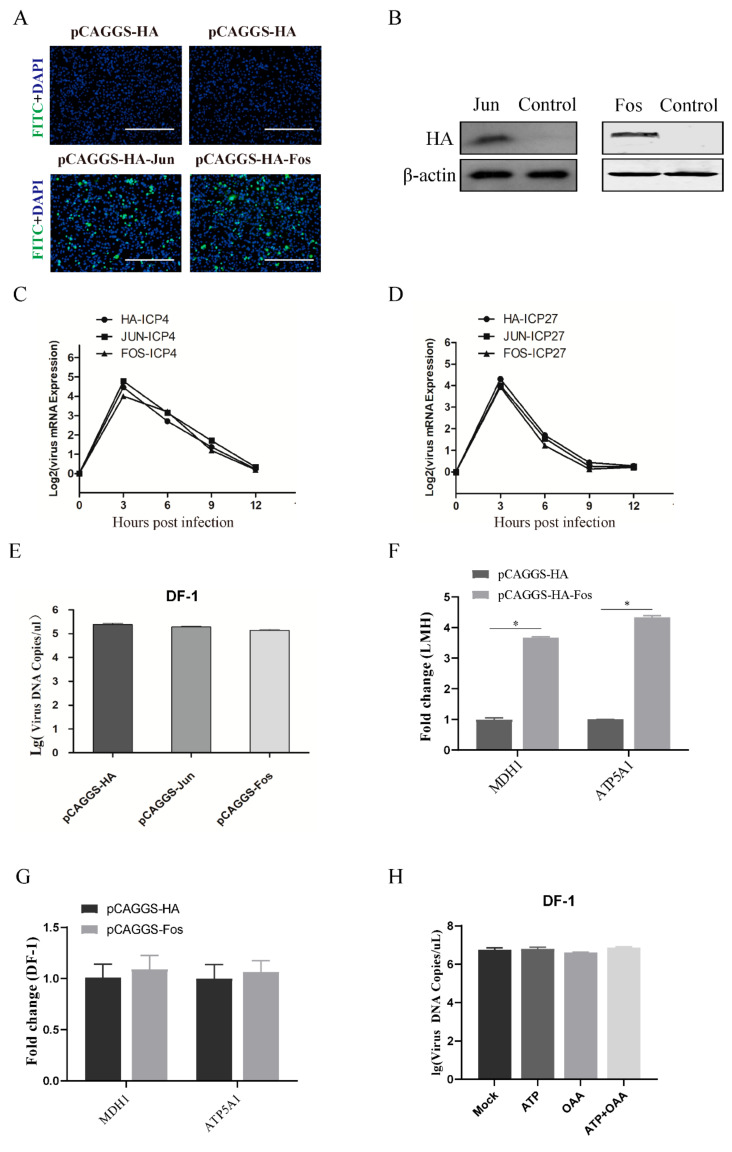
Effect of Fos/Jun overexpression on ILTV replication in DF-1 cells. DF-1 cells were transiently transfected with the pCAGGS-HA-Fos, pCAGGS-HA-Jun or pCAGGS-HA plasmid for 24 h. (**A**) Indirect immunofluorescence microscopy analysis of Fos and Jun overexpression using a mouse anti-HA antibody followed by a FITC-conjugated goat anti-mouse IgG secondary antibody (green). Chromosomes were stained with DAPI (blue). Scale bars, 150 μm. (**B**) The protein expression levels of Fos and Jun in DF-1 cells transiently transfected with the pCAGGS-HA-Fos, pCAGGS-HA-Jun or pCAGGS-HA plasmid were analyzed by Western blotting using antibodies specifically against HA (top) and β-actin (bottom). β-Actin was used as a loading control. (**C**–**E**) Twenty-four hours after transfection, DF-1 cells were infected with ILTV (MOI  =  1). The mRNA expression levels of (**C**) *ICP4* and (**D**) *ICP27* were analyzed at 12 hpi by qRT-PCR. *, *p* < 0.05. (**E**) The viral genome copy number was determined at 48 hpi by ILTV-specific qPCR. *, *p* < 0.05. (**F**,**G**) LMH cells and DF-1 cells were transfected with pCAGGS-HA (vector) or a pCAGGS-HA-Fos expression plasmid for 24 h. The transcription levels of *MDH1* and *ATP5A1* in LMH cells (**F**) and DF-1 (**G**) cells were examined at 12 hpi by qRT-PCR. (**H**) The viral genome copy number was determined by ILTV-specific qPCR in DF-1 cells pretreated with ATP (5.0 μM) and/or OAA (10 μM) at 48 hpi (ILTV, MOI = 1). The data are presented as the mean ± SD (*n* = 3). *, *p* < 0.05.

Next, we addressed whether ectopic expression of AP-1 can rescue ILTV replication in DF-1 cells. DF-1 cells were transfected with a pCAGGS-HA plasmid expressing Fos or Jun. The efficiency of overexpression was confirmed by indirect immunofluorescence and Western blot analyses using an antibody targeting HA (Figure 4A,B). Consistent with the findings in Figure 2, no effect of ectopic expression of Jun on either viral gene transcription or viral genome replication was observed in DF-1 cells (Figure 4C–E). However, overexpression of Fos also failed to rescue ILTV replication in DF-1 cells (Figure 4E). This may have been due to the lack of biological Fos function in DF-1 cells rather than the abnormal biological function of overexpressed Fos since Fos overexpression significantly improved the transcription levels of both *MDH1* and *ATP5A1* in LMH cells (Figure 4F) but not in DF-1 cells (Figure 4G). This hypothesis was confirmed by the observation that anaplerosis of neither ATP nor OAA rescued ILTV replication in DF-1 cells (Figure 4H).

### 3.5. ICP4 Is a Direct Target Gene of Fos

**Figure 5:**
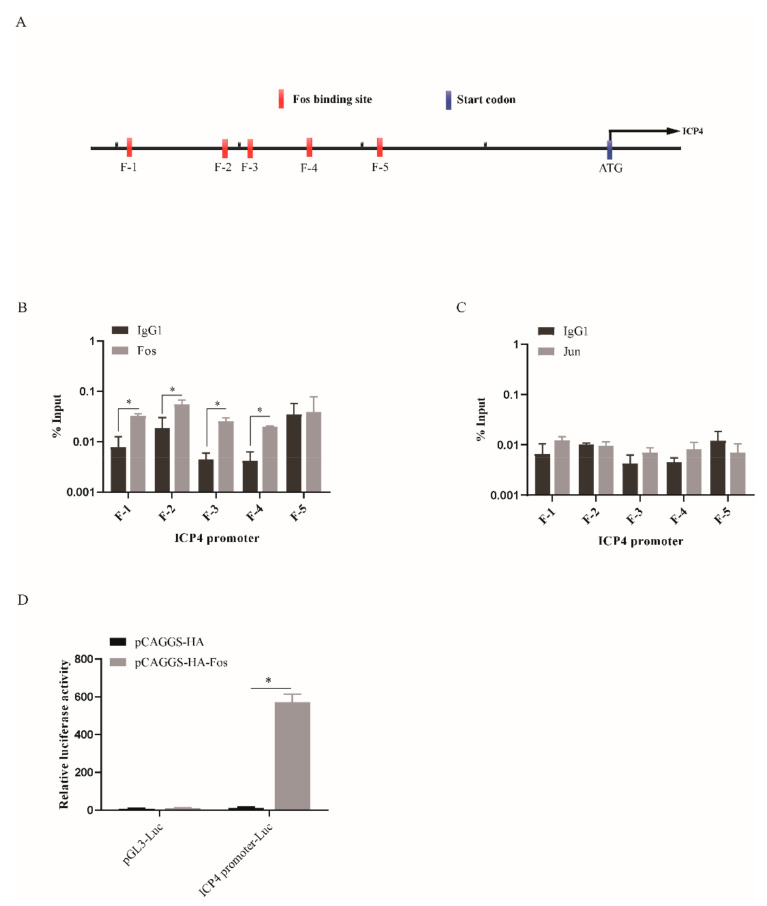
*ICP4* is a bona fide target gene of Fos. (**A**) Schematic representation of the putative binding sites of Fos (F-1, F-2, F-3, F-4, F-5) in the promoter region of *ICP4*. (**B**,**C**) LMH cells were transfected with a pCAGGS-HA (vector), pCAGGS-HA-Fos or pCAGGS-HA-Jun plasmid and harvested 12 h after infection with ILTV. ChIP assays were performed with an anti-HA antibody. IgG1 was used as a negative control. (**B**) ChIP-qPCR analysis of the amount of Fos bound to the five putative Fos-binding sites (F-1, F-2, F-3, F-4, F-5). (**C**) ChIP-qPCR analysis of the amount of Jun at the five putative Fos-binding sites (F-1, F-2, F-3, F-4, F-5). The ChIP-qPCR data were normalized to the input DNA data and are expressed as the percentage of the input. (**D**) The pCAGGS-HA and/or pCAGGS-HA-Fos plasmids were co-transfected with *ICP4* promoter-Luc and TK-*Renilla* plasmids into LMH cells. Relative luciferase activity was measured 24 h after transfection. The results are presented as fold induction after normalization to *Renilla* luciferase activity. The data are presented as the means ± SDs of three independent experiments. *, *p* < 0.05.

The viral gene transcription of ILTV is a process that is regulated by a typical cascade, and the expression of the E and L genes is under the control of the viral IE gene *ICP4* [27]. Considering the transcriptional regulation of *ICP4* by Fos (Figure 2B), we next addressed the question of whether this viral IE gene is a bona fide Fos target gene. We employed the Jaspar database version 2020 (http://jaspar.genereg.net/ (accessed on 22 January 2021)) to search for putative Fos-binding elements in the promoter of *ICP4* (Figure 5A). Five putative binding sites (F-1, F-2, F-3, F-4, and F-5) in the promoter region of *ICP4* were predicted by the program. Then, the amount of Fos bound to the putative binding sites was quantified by chromatin immunoprecipitation followed by qPCR (ChIP-qPCR) in LMH cells upon ectopic expression of Fos. Input DNA was used as a negative control, and ChIP with isotype IgG1 antibody was used to determine the level of unspecific binding of antibody to DNA. The results presented in Figure 5B demonstrate that Fos interacts with four of the five putative binding sites within the context of chromatin in living cells. Jun has been shown to bind to DNA and to regulate gene transcription frequently as a heterodimer with Fos [28]. Thus, we assessed the binding of Jun to the five putative binding sites of Fos. No interaction between Jun and any of these sites was confirmed by ChIP-qPCR in LMH cells when Jun was ectopically expressed (Figure 5C), which is consistent with our finding that knockdown of Jun has no effect on ILTV transcription and subsequent replication. To determine the biological function of the binding of Fos to the *ICP4* promoter, a dual-luciferase reporter assay was performed. The results showed that the promoter of *ICP4* upstream of the luciferase reporter gene initiated the expression of the reporter gene in a Fos-dependent manner (Figure 5D). Therefore, these data suggest that *ICP4* is a direct target gene of Fos.

## 4. Discussion

**Figure 6:**
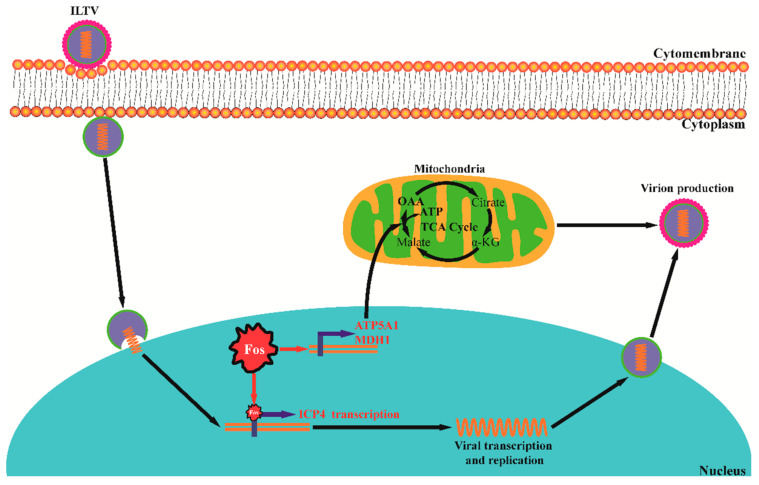
A schematic presentation of the two mechanisms by which Fos facilitates ILTV infection. On the host side, Fos maintains the supplies of OAA and ATP for viral gene transcription and subsequent processes of viral proliferation by controlling the transcription of *ATP5A1* and *MDH1*; on the virus side, Fos binds directly to the promoter of the viral IE gene *ICP4* to initiate viral transcription. The color of the arrow indicates the flux changes, and the font colors denote gene-level changes (red-direct promoted, black-no effect). α-KG: oxoglutarate, OAA: oxaloacetate.

Virus-host cell metabolic interactions are vital targets for herpesvirus infection treatment. However, to date, systematic studies of the metabolic interactions between herpesviruses and host cells have mainly focused on a few human herpesviruses. Our previous metabolomics studies illustrated the basic metabolic requirements of ILTV, which, to our knowledge, provided the first metabolic profile of animal herpesviruses [22]. Here, using the same experimental model, we further revealed host Fos as a key regulator of ILTV infection through its transcription factor functions on both the virus and host sides. As shown in Figure 6, on the host side, Fos maintains the supplies of OAA and ATP for viral gene transcription and subsequent viral proliferation by controlling the transcription of host rate-limiting enzymes; on the virus side, Fos regulates the expression of the viral IE gene *ICP4* directly through its transcription factor function.

Fos is a member of the AP-1 family of transcription factors, which are involved in many cellular and viral functions, such as cell proliferation, differentiation, cellular and viral gene expression, and tumorigenesis [29]. The expression of Fos is normally very low in quiescent cells but is easily induced by extra- and intracellular stimuli, particularly viral infections [30,31]. Studies of CMV have found that Fos disruption results in significant suppression of viral gene transcription [12]. Silencing of Fos expression has been reported to lead to a decrease in hepatitis C virus (HCV) propagation in HCV-infected cells [30]. Similar to these findings, our results showed that Fos knockdown significantly reduced viral gene expression, viral genome replication and virion production (Figure 2B–F) in ILTV-infected cells, suggesting that Fos is a key host determinant of ILTV infection.

The modulation of host metabolic enzymes has been found to be an important strategy for many human herpesviruses to replicate effectively in host cells [32]. However, the regulation of ILTV on host metabolic enzymes and its strategy remain unclear. Fos has been reported as a key regulator of many rate-limiting enzymes of the metabolic synthesis pathway [31]. In the present study, two metabolic genes, *ATP5A1* and *MDH1*, were found to be suppressed by Fos knockdown at the transcriptional level in ILTV-infected cells (Figure 3C). MDH1 catalyzes the reversible oxidation of L-malate to an important TCA cycle intermediate OAA, and ATP5A1 is the rate-limiting enzyme of the synthesis of ATP from ADP and inorganic phosphate [26]. Our previous study revealed insufficient production of both OAA and ATP in DF-1 cells upon ILTV infection [22]. Here, we found that the anaplerosis of both OAA and ATP completely rescued the reduction in *ICP4* transcription (Figure 3E) and the subsequent reduction in virion production (Figure 3F) in Fos-silenced LMH cells. Thus, it is possible that Fos regulates ILTV replication by maintaining host OAA and ATP supplies via its transcriptional control of *MDH1* and *ATP5A1*. However, neither overexpression of Fos nor anaplerosis of ATP and/or OAA rescued ILTV replication in the susceptible but nonpermissive DF-1 cell line (Figure 4). This was not due to the abnormal biological function of Fos overexpression since the overexpression of Fos significantly improved the transcription of both *MDH1* and *ATP5A1* in LMH cells (Figure 4F) but not in DF-1 cells (Figure 4G). It is possible that the biological function of Fos is cell type-specific and is lacking in DF-1 cells due to the absence of Fos cofactors. In view of the lack of corresponding antibodies in the chicken, it would be best to evaluate the proteome after Fos immunoprecipitation to identify possible interaction partners of Fos, which will be conducted later to elucidate the exact mechanisms of its functions.

Furthermore, direct interactions between Fos and viral genes have been observed in human cells infected with KSHV [12]. A recent study revealed that ORF45-mediated prolonged Fos accumulation promotes viral transcription and lytic replication during the lytic infection of KSHV through direct binding of Fos to the promoter of the KSHV gene *ORF50* [13]. In the present study, we found that Fos binds directly to the promoter of the IE gene of ILTV, *ICP4*, to regulate its transcription. Similar to that of other herpesviruses, the viral gene transcription of ILTV is regulated by a typical cascade [27]. *ICP4*, the only known IE gene of ILTV, is the initial regulator of this cascade [33,34,35]. Our findings indicate that Fos may regulate the ILTV transcription cascade and subsequent ILTV replication by direct regulation of ILTV IE gene transcription.

## 5. Conclusions

In summary, we identified Fos as a key host regulator of ILTV infection and revealed two mechanisms by which Fos facilitates ILTV infection: on the virus side, Fos binds directly to the promoter of the viral IE gene *ICP4* to initiate viral transcription; on the host cell side, Fos maintains the supplies of OAA and ATP for viral gene transcription and subsequent processes of viral proliferation through controlling the transcription of rate-limiting enzymes (Figure 6). Our study may improve the current understanding of both avian herpesvirus-host interactions and the roles of AP-1 in viral infection.

## Figures and Tables

**Table 1:** Primer sequence for construct plasmid.

Primer Name	Sequence (5′-3′)	Restriction Recognition Sites	Length (bp)
Fos F	GGGGTACCATGTACCAGGGCTTCGCTGGGG	*KpnI*	1101
Fos R	CCGCTCGAGCAAGGCCAGCAGGGTGGGGGAGC	*XhoI*	
Jun F	GGGGTACCGAGCCTACTTTCTACGAGGATGC	*KpnI*	945
Jun R	CCGCTCGAGAAACGTTTGCAACTGTTGTGTTAGC	*XhoI*	
ICP4 F	TTTCTCTATCGATAGGTACCGACTTCCTTGGCACTCCCAG	*KpnI*	1001
ICP4 R	GATCGCAGATCTCGAGGATAGAATGCTAGTGGAAGGACTAG	*XhoI*	
ICP27 F	TTTCTCTATCGATAGGTACCGATCACTGATGGGTTACACGACTCC	*KpnI*	1000
ICP27 R	GATCGCAGATCTCGAGTCGAACAGCGACGGCGCC	*XhoI*	

The restriction enzyme sites in primer sequences are underlined.

**Table 2:** Primer sequence for qRT-PCR.

Primer Name	Sequence (5′-3′)	Length (bp)
ICP4 F	AATGCCATACCCAAACCAACT	174
ICP4 R	AATGACAGCCCACCTAATCCC	
ICP27 F	GGTTGGCAAAAATTTCACGAG	180
ICP27 R	GATGCATGGCTAGAGATTGAC	
gC F	AAATGCTACGACCTGAAACT	203
gC R	CTCGGGCTCATCCAAAACA	
β-actin F	GTGGATCAGCAAGCAGGAGT	151
β-actin R	ATAAAGCCATGCCAATCTCGT	
Fos F	GTGAGAGCTGGTAGTCTGT	185
Fos R	ATATTGCCAGGAACACAGTAG	
Jun F	CAGCATCACATAAACCCCCAG	160
Jun R	TCATGCGTTTTCTCTCGGCTT	
TPI1 F	GAGAAGCTGGATGAGAGAGA	147
TPI1 R	AGTAGCAGTTTTACCAGTTCC	
MDH1 F	AGTCAGCCCCATCAATACCA	154
MDH1 R	GGATACTGAGTGGAGGAGTGG	
MDH2 F	AGGGAGAATTCAAGAAGCTGG	181
MDH2 R	GTATGGGCTCTCCGTCTCTTC	
LDHB F	AACATGGTGATTCTAGTGTGG	172
LDHB R	TTCGTATACCCCTTGAGTCTG	
ADK F	TTTTGGAAATGAGACGGAAG	159
ADK R	ATTACAGTGTCTTCTTTCCCC	
ATP5A1 F	TCTGTGTCCCGCGTAGGTTCTG	168
ATP5A1 R	TGTCAGACGCACGCCACGATT	
ATP5C1 F	GAGATGCTTCAGTCATTGCTT	160
ATP5C1 R	ATACTCAGGCTTTCAGAACCA	
IDH3A F	TGAGCATGTGATTGTTGAAGG	170
IDH3A R	TCAAGAAAAGCCCATCAGACA	

**Table 3:** Primers for ChIP-qPCR of IE genes *ICP4* promoter of ILTV.

Primer Name	Sequence	Length (bp)
4-1 F	ACTTCCTTGGCACTCCCAGGAA	200
4-1 R	CTTGTGTCCACAGGTACGTGCAA	
4-2 F	TCAAAATAAACTTCACTGCTT	200
4-2 R	CCTGAGCCTACTGTAGAACA	
4-3 F	GGCTCAGGTTTGTTTATTATGT	138
4-3 R	GTTAGCGAGCGTATTTCCAGT	
4-4 F	AATACGCTCGCTAACGATATG	156
4-4 R	TGGGTAGGTTTCAGCAAAGG	
4-5 F	CACCTTTGCTGAAACCTACCC	479
4-5 R	AATCCTGCAGAACAAGACCC	

## Data Availability

The data presented in this study are available on request from the corresponding author.

## Abbreviations

ILTV: avian infectious laryngotracheitis virus; AP-1: activator protein 1; TF: transcription factor; KSHV: Kaposi’s sarcoma-associated herpes virus; CMV: cytomegalovirus; HSV-2: herpes simplex virus type 2; HCV: hepatitis C virus; MDV: Marek’s disease virus; IE: immediate-early; E: early; L: late; LMH: leghorn male hepatoma; DMEM: Dulbecco’s modified eagle’s medium; FBS: fetal bovine serum; ChIP: chromatin immunoprecipitation; DEGs: differently expressed genes; TFBS: transcription factor binding site; CPE: cytopathogenic effect; MDH: malate dehydrogenase; OAA: oxaloacetate.
